# Towards One Health surveillance of antibiotic resistance: characterisation and mapping of existing programmes in humans, animals, food and the environment in France, 2021

**DOI:** 10.2807/1560-7917.ES.2023.28.22.2200804

**Published:** 2023-06-01

**Authors:** Lucie Collineau, Clémence Bourély, Léo Rousset, Anne Berger-Carbonne, Marie-Cécile Ploy, Céline Pulcini, Mélanie Colomb-Cotinat

**Affiliations:** 1 University of Lyon, French Agency for Food, Environmental and Occupational Health & Safety (ANSES), Epidemiology and Surveillance Support Unit, Lyon, France; 2 French Ministry of Agriculture and Food Sovereignty, General Directorate for Food, Animal Health Unit, Paris, France; 3 Claude Bernard University Lyon 1, Lyon, France; 4 VetAgro Sup, Marcy L’Etoile, France; 5 Direction des maladies infectieuses, Santé Publique France, Saint-Maurice, France; 6 Université de Limoges, INSERM, CHU Limoges, UMR 1092, Limoges, France; 7 French Ministry for Health and prevention, Paris, France; 8 CHRU-Nancy, Université de Lorraine, Nancy, France; 9 Université de Lorraine, APEMAC, Nancy, France

**Keywords:** Surveillance, epidemiology, antimicrobial resistance, ABR, AMR, antimicrobial use, antibiotic residues, mapping, integration, One Health

## Abstract

**Background:**

International organisations are calling for One Health approaches to tackle antimicrobial resistance. In France, getting an overview of the current surveillance system and its level of integration is difficult due to the diversity of surveillance programmes.

**Aim:**

This study aimed to map and describe all French surveillance programmes for antibiotic resistance (ABR), antibiotic use (ABU) and antibiotic residues, in humans, animals, food and the environment, in 2021. Another objective was to identify integration points, gaps and overlaps in the system.

**Methods:**

We reviewed the literature for surveillance programmes and their descriptions. To further characterise programmes found, semi-directed interviews were conducted with their coordinators.

**Results:**

In total 48 programmes in the human (n = 35), animal (n = 12), food (n = 3) and/or the environment (n = 1) sectors were identified; 35 programmes focused on ABR, 14 on ABU and two on antibiotic residues. Two programmes were cross-sectoral. Among the 35 ABR programmes, 23 collected bacterial isolates. Bacteria most targeted were *Escherichia coli* (n = 17 programmes), *Klebsiella pneumoniae* (n = 13), and *Staphylococcus aureus* (n = 12). Extended-spectrum beta-lactamase-producing *E. coli* was monitored by most ABR programmes (15 of 35) in humans, animals and food, and is a good candidate for integrated analyses. ABU indicators were highly variable. Areas poorly covered were the environmental sector, overseas territories, antibiotic-resistant-bacterial colonisation in humans and ABU in companion animals.

**Conclusion:**

The French surveillance system appears extensive but has gaps and is highly fragmented. We believe our mapping will interest policymakers and surveillance stakeholders. Our methodology may inspire other countries considering One Health surveillance of ABR.

**Key public health message**

**What did you want to address in this study?**

Antibiotic resistance (ABR) threatens the successful treatment of bacterial infections. It can develop when bacteria are exposed to antibiotics in people, animals, and the environment. To tackle ABR, international/European organisations have called for a One health approach. To this end, in France, we first needed to get an overview of the surveillance system for ABR, and its constitutive programmes in the human, animal and environmental sectors.

**What have we learnt from this study?**

In 2021, 48 programmes were found to contribute to the French ABR surveillance system. The programmes relied on several types and sources of data. They monitored various bacterial species, and antibiotics’ use, antibiotic residues’ occurrence or ABR in different human or animal populations, in food and in the environment. While resourceful, the surveillance system appeared complex and lacked integration across sectors and hazards.

**What are the implications of your findings for public health?**

This study represents a first step towards One Health surveillance of antibiotic resistance in France. Based on its findings we recommend increasing common ways to measure antibiotic use, resistance and residues across programmes and integrated data analyses across human, animal, food and/or the environment sectors. Our approach can easily be reproduced in other settings and will likely inspire other countries considering One Health surveillance.

## Introduction

Antibiotic resistance (ABR) is a threat to modern healthcare and is recognised as a major public health problem [1,2]. Since antibiotic-resistant microorganisms can occur in humans, animals, food and in diverse ecosystems, prevention of ABR is a complex issue that, in order to be addressed, calls for integrated policies at the human–animal–environment interface. In this regard, the Word Health Organization (WHO) published in 2015 a Global Action Plan on Antimicrobial resistance, which underscored the need for a One Health approach to ABR surveillance [3]. This plan was jointly adopted by the World Organisation for Animal Health and the Food and Agriculture Organization of the United Nations. As for Europe, the 2017 European One Health Antimicrobial Resistance Action Plan [4] also argued for a more integrated ABR surveillance system, which would also monitor antibiotic use (ABU), as well as antibiotic residues in ecosystems. By 2021, most European countries had already set up mandatory or voluntary programmes for surveillance of ABR in humans [5], companion and food-producing animals and food [6]. Moreover, the regular joint inter-agency reports on integrated analysis of antimicrobial agent consumption and occurrence of antimicrobial resistance in bacteria from humans and food-producing animals (JIACRA), which continue to date, contributed to a better understanding of ABR and provided valuable insights for policymakers [7].

In France, numerous surveillance programmes relating to ABR are currently in place. These cover ABR and ABU in both humans and animals, as well as antibiotic residues in food and in the environment. The 2016 inter-ministerial roadmap for controlling antimicrobial resistance [8] conveyed a will to make surveillance data more usable and efficiently sharable across the human, animal/food and environmental sectors and to promote cross-sectoral collaborations; these initiatives would complement other activities defined in the sectorial national action plans [9-11]. Nevertheless, the large number and diversity of surveillance programmes in the country make it difficult to obtain an exhaustive picture of the surveillance system. Its comprehensive mapping is therefore an essential prerequisite to evaluate and facilitate collaborations between the programmes.

Hence, the aim of this study was to identify, map and characterise all French surveillance programmes for ABR, ABU and antibiotic residues existing in humans, animals, food and the environment, and to identify integration points, gaps and overlaps.

## Methods

### Inclusion and exclusion criteria

As the French surveillance initiatives were highly variable in terms of geographic scope, objectives and sustainability, we retained for analysis only those corresponding to the following definition of a surveillance programme: ‘a structured group of actors and/or institutions in charge of collecting, centralising, analysing and communicating quantitative data on a regular and long-term basis’ [12]. Both local/regional and national surveillance programmes were included. Exclusion criteria were unrepeated research studies, inactive programmes at the time of the literature review, clinical research programmes, as well as programmes assessing appropriateness of antibiotic use. The focus was on antibiotics only, excluding other antimicrobials.

### Identification of surveillance programmes

A literature review was conducted in January–February 2021 in both the scientific and grey literatures (in English and French languages), to identify all potential French programmes for surveillance of ABR, ABU and antibiotic residues in humans, animals, food and the environment. Primary literature sources were official websites of ministries, public health agencies, and other public and private institutions involved in ABR-related surveillance. To screen the scientific literature, the following search string was used in PubMed, including articles published since 2005 only: (antimicrobial*[Title/Abstract] OR antibiotic*)[Title/Abstract] AND (surveillance[Title/Abstract] OR monitoring)[Title/Abstract] AND France[Title/Abstract]. After listing all potential programmes identified, the coordinator of each programme was contacted by email to check if the programme matched the inclusion criteria. Lastly, the list of identified programmes was submitted to a group of 19 French experts with long-term expertise in surveillance or policy-making related to ABR, ABU and antibiotic residues surveillance in the human, animal, food and environmental sectors, in order to identify any potential missing programme and validate the final list.

### Characterisation of surveillance programmes and mapping

Surveillance programmes were characterised using a standardised grid adapted from the ECoSur matrix developed by Bordier et al. [13]. The grid included 28 variables of interest covering aspects related to organisation (e.g. regulatory status, ownership, steering and coordination activities), methods and operations (e.g. target population, coverage, sampling strategy, data collection and analysis, indicators used, dissemination of the results). Contribution to supra-national surveillance programmes was also recorded. The detailed descriptive grid is provided in Supplementary material S1 (Table S1; List and characterisation of the 48 programmes retained in the study).

For each included surveillance programme, the descriptive grid was pre-completed using information collected from the literature by four members of the research team, including two scientists from the human sector and two from the animal sector. Subsequently, semi-directed interviews with the programmes’ coordinators were conducted by the research team using an interview guide (Supplementary material S2; Guide for interviews) to complete and validate the grid. Interviews were performed between February and June 2021, using online videoconferencing because of COVID-19 related restrictions. Lastly, based on collected data, a visual representation was produced to display the mapping of the surveillance system and make it easier to identify integration points across sectors, as well as overlaps and gaps.

## Results

### Selection process and data collection

Of the 79 surveillance initiatives initially identified, 48 matched our inclusion criteria and were included for further analysis (Table S2 in supplementary material; List of all 79 initiatives initially identified and reasons for exclusion). A total of 36 interviews with programme coordinators were conducted to collect information on 40 programmes. For the remaining eight programmes, information was validated via email exchange.

### Sectors, populations and targets

Of the 48 included programmes, 35 targeted the human, 12 the animal, three the food and one the environmental sectors (Table 1). Two programmes were cross-sectoral, and covered both the human and animal or food sectors (Figure). In the 35 human sector programmes, some were part of networks or larger organisations (i.e. subsystems). Seven national programmes belonged to the French network for prevention of healthcare-associated infections and ABR (RéPias), launched in 2018 and led by Santé publique France, the French public health agency. The Répias is a key support to the national strategy for preventing infections and ABR in the human sector; it produces surveillance data on healthcare associated infections, ABR and antibiotic consumption, and supports infection prevention and control tools and public health communication media [14]. In addition, the French National Observatory for Epidemiology of Bacterial Resistance to Antimicrobials (ONERBA), existing since 1997, grouped together 10 voluntary programmes (nine in the human and one in the animal sector). Of these, four were bacterial species-specific (Table S1), and two had regional coverage [15]. Last, 16 were national reference centres (NRCs) coordinated by Santé publique France, with two of these also operating under ONERBA.

**Table 1:** Distribution of surveillance programmes according to sector, population and target, France, 2021 (n = 48 programmes)^a^

Sector (number of programmes)^a^	Population (number of programmes)	Number of programmes covering the target of interest^b^ (IDs of corresponding programmes)		
		ABR (n = 35)	ABU (n = 14)	Residues (n = 2)
Human (n = 35)^a^	Healthcare facilities (n = 30)	29 (1–17, 19, 21, 23, 24, 28, 32–36, 44, 46)	3 (18, 21, 44)	NA
	Community (n = 23)	19 (1, 3–16, 19, 24, 35, 45)	4 (18, 37, 39, 48)	NA
	Long-term care facilities (n = 20)	18 (3–16, 20, 24, 35, 45)	3 (18, 20, 37)	NA
Animal (n = 12)^a^	Diseased food-producing animals (n = 10)	3 (25, 30, 31)	7 (22, 27, 38, 40–43)	NA
	Diseased companion animals (n = 2)	1 (30)	1 (43)	NA
	Healthy food-producing animals (n = 2)	2 (19, 26)	None	NA
Food (n = 3)^a^	Food of animal and non-animal origin (n = 1)	1 (11)	None	None
	Food of animal origin (n = 2)	1 (26)	None	1 (29)
Environment (n = 1)	Surface and ground water (n = 1)	None	None	1 (47)

ABR: antibiotic resistance; ABU: antibiotic use; ID: identifier; NA: not applicable.

^a^ A programme may target more than one sector, population or target.

^b^ The correspondence between IDs and programme names is described in the Supplementary material (Table S1).

**Figure:**
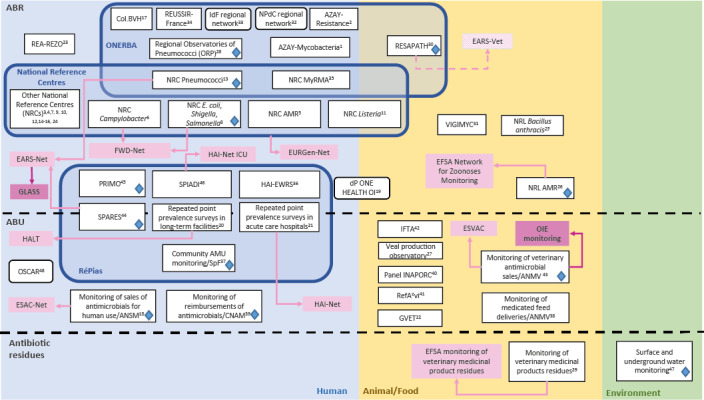
Mapping of the existing surveillance programmes for antibiotic resistance (ABR), antibiotic use (ABU) and antibiotic residues in humans, animals/food and the environment in France in 2021 (n = 48 programmes)

ABR: antibiotic resistance; ABU: antibiotic use; EARS-Net: European Antimicrobial Resistance Surveillance Network; EFSA: European Food Safety Authority; ESAC-Net: European Surveillance of Antimicrobial Consumption Network; ESVAC: European Surveillance of Veterinary Antimicrobial Consumption; EURGen-Net: European Antimicrobial Resistance Genes Surveillance Network; FWD-Net: European Food and Waterborne Diseases and Zoonoses Surveillance Network; GLASS: Global Antimicrobial Resistance and Use Surveillance System; HAI-Net: Healthcare-Associated Infections Network; HAI-Net ICU: HAI-Net surveillance of Healthcare-Associated Infections in Intensive Care Units; HALT: Healthcare Associated Infections in Long Term Care Facilities; NRL: national reference laboratory; OIE: World Organisation for Animal Health; ONERBA: French National Observatory for Epidemiology of Bacterial Resistance to Antimicrobials; Répias: French network for prevention of healthcare-associated infections and ABR; NRCs: national reference centres.

In the Figure, the numbers in superscript represent the identifiers (IDs) of the programmes (Table S1).

Among the 48 programmes, the majority (n = 35) focused on ABR (Table 1). Three programmes in the human sector monitored both ABR and ABU. All 31 human ABR-related programmes collected data from clinical samples; two programmes also collected data from screening samples (colonisation). In the animal sector, most programmes focused on diseased or healthy food-producing animals (n = 12) and targeted multiple animal species (n = 7), horses (n = 3), pigs (n = 2), poultry (n = 1), veal calves (n = 1) or rabbits (n = 1). Two programmes targeted diseased companion animals, including dogs (n = 2) and cats (n = 2). The environmental programme targeted surface and ground water.

### Regulatory status, funding and durability

The French surveillance system relied mainly on public funds, as 34 of 48 programmes were solely publicly funded. Nine programmes relied on mixed public–private funding, and five were privately funded (Table S1; List and characterisation of the 48 programmes retained in the study). A total of 29 surveillance programmes were regulated (coordinated by authorities but implemented by other actors) or official (coordinated and implemented by authorities), although 19 programmes were independently run by voluntary actors. Most programmes (n = 27) were established before 2010, and 15 were built within the past 10 years, of which nine in the last 5 years. For six programmes, the creation date could not be retrieved.

### Timeliness, geographic coverage and granularity

Most programmes (n = 24) collected data throughout the year without interruption, although some programmes collected data annually (n = 14), infra-annually (n = 6) or multi-annually (e.g. every 3 years, n = 3); frequency of data collection was missing for one programme. Dissemination of the results occurred on an annual (n = 43), pluri-annual (n = 3) or infra-annual (n = 2) basis. Of 48 programmes, 16 also disseminated their results via open-access dashboards. Most programmes had national coverage (n = 43) and among them, 31 included at least one overseas territory (with some overseas territories, such as Réunion or Guadeloupe, covered by several national programmes, others not at all). Among the 43 nationwide programmes, all displayed their results at a national level, but 10 programmes with a higher granularity also displayed their results at a sub-national level. In addition, five regional programmes displayed their results at a single regional level only.

### Surveillance design and data collected

The majority (41/48) of surveillance programmes relied on passive surveillance. Among the 35 ABR surveillance programmes, 23 had access to bacterial isolates and were able to perform molecular characterisation (e.g. PCR or whole-genome sequencing) on all or part of the collected isolates in addition to conventional antibiotic susceptibility testing (Table 2). Concerning the 14 ABU programmes, surveillance was primarily based on administration (n = 6), deliveries/dispensing data (n = 5), reimbursements (n = 3), sales data (n = 2), or prescriptions (n = 1), noting that a programme could provide more than one type of ABU data.

**Table 2:** Distribution of surveillance programmes according to data collected, France, 2021 (n = 48 programmes)^a^

Target	Number of programmes^a^	Data collected (n = number of programmes)	Corresponding IDs^b^
ABR	35	Resistance data^c^ only (n = 12)	2, 17, 20, 21, 23, 32–34, 36, 44–46
		Resistance data and bacterial isolates (n = 23)	1, 3–16, 19, 24–26, 28, 30, 31, 35
ABU	14	Administration (n = 6)	20–22, 27, 41, 42
		Deliveries or dispensing (n = 5)	27, 38, 40, 41, 44
		Reimbursements (n = 3)	37, 39, 48
		Sales (n = 2)	18, 43
		Prescriptions (n = 1)	41
Residues	2	Residues data (n = 2)	29, 47

ABR: antibiotic resistance; ABU: antibiotic use; ID: identifier; SIR: susceptible, intermediate, resistant.

^a^ A programme may collect several types of data.

^b^ The correspondence between IDs and names of the programme is described in the Supplementary material (Table S1).

^c^ Resistance data include either quantitative (minimum inhibitory concentrations or disk diffusion diameters) or qualitative (SIR profiles) data from antibiotic susceptibility testing, without further access to bacterial isolates.

### Targeted bacteria

Among the 35 programmes monitoring ABR in humans or animals, 19 covered multiple bacterial species simultaneously, while 16 targeted a single bacterial species (Table S1; List and characterisation of the 48 programmes retained in the study); typically these programmes were run by NRCs (n = 16). Bacterial species of primary interest were *Escherichia coli* (n = 17 programmes), *Klebsiella pneumoniae* (n = 13), *Staphylococcus aureus* (n = 12). More specifically, extended-spectrum beta-lactamases (ESBLs)-producing *E. coli* were monitored by 15 programmes and meticillin-resistant *Staphylococcus aureus* (MRSA) by 12 programmes (Table 3).

**Table 3:** Bacteria species and resistance phenotypes most monitored in the human, animal and food sector, France, 2021 (n = 48 programmes)

Bacteria species	Resistance phenotype of interest (n = number of programmes)	Number of programmes (corresponding IDs)^a^		
		Human sector	Animal sector	Food sector
*Escherichia coli*	All (n = 17)	15 (2, 5, 8, 17, 19–21, 23, 32–34, 36, 44–46)	3 (19, 26, 30)	1 (26)
	ESBL producing^b^ (n = 15)	14 (2, 5, 8, 17, 19–21, 23, 32–34, 44–46)	1 (26)	1 (26)
	Carbapenemase producing^b^ (n = 15)	14 (2, 5, 8, 19–21, 23, 32–34, 36, 44–46)	1 (26)	1 (26)
	Fully susceptible (n = 2)	0	2 (26, 30)	1 (26)
*Klebsiella pneumoniae*	All (n = 13)	12 (2, 5, 17, 20, 21, 23, 33, 34, 36, 44–46)	1 (30)	0 (NA)
	ESBL-producing^b^ (n = 10)	10 (2, 5, 17, 21, 23, 33, 34, 44–46)	0 (NA)	0 (NA)
	Carbapenemase-producing^b^ (n = 10)	10 (2, 5, 21, 20, 33, 34, 36, 44–46)	0 (NA)	0 (NA)
*Staphylococcus aureus*	All (n = 12)	11 (2, 14, 17, 20, 21, 23, 33, 34, 44–46)	1 (30)	0 (NA)
	MRSA^b^ (n = 12)	11 (2, 14, 17, 20, 21, 23, 33, 34, 44–46)	1 (30)	0 (NA)
*Enterococcus faecium* or *E. faecalis*	All (n = 13)	11 (2, 5, 17, 20, 21, 23, 33, 34, 36, 44, 46)	2 (26, 30)	0 (NA)
*Pseudomonas aeruginosa*	All (n = 12)	11 (2, 5, 17, 20, 21, 23, 32, 33, 34, 44, 46)	1 (30)	0 (NA)
	Carbapenem-resistant (n = 10)	10 (2, 5, 17, 20, 21, 23, 33, 34, 44, 46)	0 (NA)	0 (NA)
*Acinetobacter baumannii*	All (n = 7)	6 (5, 20, 21, 23, 44, 46)	1 (30)	0 (NA)
	Carbapenem-resistant (n = 6)	6 (5, 20, 21, 23, 44, 46)	0 (NA)	0 (NA)
*Salmonella enterica*	All (n = 3)	2 (5, 8)	1 (26)	1 (26)
*Campylobacter* spp.	All (n = 2)	1 (6)	1 (26)	1 (26)

ESBL: extended-spectrum beta-lactamase; ID: identifier; MRSA: meticillin resistant *Staphylococcus aureus*; NA: not applicable; 3GC: third-generation cephalosporins.

^a^ A programme may collect several types of data and serve different sectors. The correspondence between IDs and programme names is described in the Supplementary material (Table S1).

^b^ Programme for which ESBL or carbapenemase production or meticillin resistance is confirmed by routine using techniques recommended in France.

### Indicators of ABR, ABU and antibiotic residues

A large majority of ABR-related programmes monitored proportion of resistant isolates (33/35), although a few programmes worked with different indicators, including the incidence rate (n = 4) or number of cases (n = 2) of infections with ABR bacteria, as well as the prevalence of samples harbouring at least one ABR isolate (n = 1). Different standards were used to determine resistance profiles: all 31 human-related programmes were using clinical breakpoints defined by the European Committee on Antimicrobial Susceptibility Testing (EUCAST), animal-related programmes were using epidemiological cut-off values (ECOFFs) from either EUCAST (n = 2) or the veterinary section of the Antibiogram Committee of the French Society of Microbiology (CASFM) (n = 1), or clinical breakpoints from the Clinical and Laboratory Standards Institute (CLSI) (n = 1) or simply providing minimum inhibitory concentration distributions in the absence of available interpretation criteria (n = 1, *Mycoplasma* spp. in ruminant animals). The indicators for ABU surveillance were highly variable both within and between the human and animal sectors (Table 4) and depending on the targeted population. Detailed description of ABU indicators calculation has been provided elsewhere [16].

**Table 4:** Distribution of surveillance programmes according to antibiotic use indicators, France, 2021 (n = 14 programmes)^a^

Sector and sub-sector	Number of programmes	Indicator	Number of programmes	Corresponding IDs^b^
Human				
All sub-sectors	7	Variable	7	18, 20, 21, 37, 39, 44, 48
Healthcare facilities	3	DDD/1,000 hospitalisation days	1	44
		DDD/1,000 inhabitants/day	1	18
		Prevalence of treated patients/100 hospitalised patients	1	21
Community	3	DDD/1,000 inhabitants/day	2	37, 48
		Prescriptions/1,000 inhabitants/day	1	37
		Number of treatments/100 outpatients^c^	1	39
Long-term care facilities	3	DDD/1,000 residents/day	1	18
		Prescriptions/1,000 residents/day	1	37
		Prevalence of treated residents/100 residents	1	20
Animal				
All sub-sectors	7	ALEA	6	22, 27, 38, 40, 41, 43
		Treatment days/animal	3	22, 27, 40
		Treatments/animal	3	22, 27, 40
		Tons of antibiotics sold/year	2	38, 43
		Live weight treated (nb-ACD, nb-DCDvet)	2	38, 43
		Amount of active substance/biomass at risk (mg of active substance /PCU)	1	43
		Live weight daily treated (nb-ADD, nb-DDDvet)	1	43
		DDD/kg slaughtered	1	41
		IFTA	1	42

ALEA: animal level of exposure to antimicrobials, corresponding to the ratio between the estimated live weight treated and the biomass of the animal population; DDD: defined daily dose; ID: identifier; IFTA: index of frequency of treatments with antibiotics, corresponding to the ratio between the number of days treated and the duration of the production period; nb-ACD and nb-DCDvet: number of animal course doses based on national (ACD) or European (DCDvet) standards; nb-ADD and nb-DDDvet: number of animal daily doses based on national (ADD) or European (DDDvet) standards; PCU: population correction unit.

^a^ A programme may use several types of indicators.

^b^ The correspondence between IDs and names of the programme is described in the Supplementary material (Table S1).

^c^ Only for patient aged from 16 to 65 years old, without chronic disease.

Regarding antibiotic residues surveillance, the indicator used in one food programme was the proportion of samples beyond the maximum residue level (MRL) for sulfonamides and quinolones [17], while the indicator used in the environment was the proportion of samples beyond the ‘predicted no effect concentration’ (PNEC) for macrolides, fluoroquinolones and sulfonamides–diaminopyrimidines [18].

### Contribution to supranational surveillance programmes

In addition to supporting national initiatives against ABR, the French surveillance programmes contributed to 10 European and two international established programmes for surveillance of ABR, ABU, or antibiotic residues (Figure), and to one programme under construction for ABR surveillance in diseased animals (EARS-Vet) [19].

### Gaps and overlap

The characterisation of objectives and targets of each monitoring programme allowed us to identify some gaps and an overlap in the French surveillance system (Box). Some areas appeared to be insufficiently covered by the current surveillance system: the environmental sector, overseas territories, ABU in companion animals, ABR in non-captive wild animals. Conversely, in the human sector, five programmes targeted ABR in healthcare facilities.

Box **List of the gaps and overlap identified in the coverage of the surveillance systems for antibiotic resistance, antibiotic use and antibiotic residues, France, 2021**

**Gaps**

• Lack of structured national surveillance programmes in the environmental sector.

• Antibiotic residues only routinely monitored in surface water and animal-derived food.

• Overseas territories poorly represented.

• ABR surveillance in the human sector mostly targeting clinical samples, and rarely screening samples.

• Lack of a dedicated ABU-surveillance programme in companion animals.

• Lack of ABR surveillance in non-captive wild animals and aquaculture.

• Lack of ABR testing in diseased animals to antibiotics of primary interest in human health (e.g. carbapenems), since routine testing is limited to antibiotics authorised in veterinary medicine.

**Overlap**

• Five programmes targeted ABR data collection in healthcare facilities in the human sector.

ABR: antibiotic resistance; ABU: antibiotic use.

### Discussion

The present study provided the first comprehensive overview of the French surveillance system for ABR, ABU, and antibiotic residues, including a mapping and characterisation of the 48 surveillance programmes existing in 2021 in humans, animals, food, and the environment, as well as the identification of major gaps and overlaps. For comparison, 11 programmes for ABR/ABU surveillance were identified in the United Kingdom (UK) in 2019 [20] and 29 in Canada in 2020 including six national, 22 provincial and one territorial programme [21]. The large number of French programmes stems from several factors: (i) phenotypic and molecular ABR data were collected through separate surveillance programmes, (ii) NRCs for humans and national reference laboratories (NRLs) for animals were split by bacterial species, and (iii) historically, ABU surveillance programmes in the animal sector have monitored separate animal species (i.e. with each sector/industry having developed its own programme).

Despite their large number, the French programmes complemented each other by targeting different populations and providing evidence to support and evaluate national actions [9-11]. Moreover, most programmes produced surveillance reports at least annually. This yearly reporting is commonly used to support operational surveillance of the ABR epidemiological situation and to guide prevention and control strategies. Nevertheless, a recent study within the scope of the European joint action on antimicrobial resistance and healthcare associated infections (EU-JAMRAI) reported the feasibility of quarterly reporting in Europe, to timely inform interventions at local, regional, and national levels [22]. We showed that in France, very few programmes are currently reporting on an infra-annual basis, and this should be considered as a room for improvement. Several programmes also contributed to supra-national programmes, enabling the French surveillance system to respond to European and international requirements.

On the other hand, the French surveillance system appeared fragmented, as most surveillance programmes were addressing a single sector and focused on either solely ABR or ABU. Only three programmes, all in the human sector, targeted both ABR and ABU. Moreover, only two programmes covered both human and animal sectors, with one programme at sub-national level. Like in France, the UK surveillance system appeared fragmented with limited integration between surveillance programmes [20]. In contrast, the Canadian ABR Surveillance System appeared at an advanced stage of integration, although surveillance coverage was incomplete and highly variable between provinces/territories [21].

In France, two subsystems partly counterbalanced the apparent lack of integration, by making collaborations between programmes easier. The RéPias contributed to integrate ABU and ABR monitoring in the human sector, while ONERBA facilitated ABR surveillance integration between the human and animal sectors. In addition, a joint One Health Antibiotic Resistance brochure [23], led by Santé publique France and gathering 12 programmes from the three human, animal/food and environment sectors, is published each year during the World Antimicrobial Awareness Week in November [24]. It appears as an integrative effort towards One Health across sectors and targets, although limited to joint external communication of the results produced independently by each programme. An additional working group facilitating integrated analyses across sectors, inspired from JIACRA but based on French-specific data, would nicely complement this activity.

Indeed, the need for integrated analysis of surveillance data from the three sectors has been re-emphasised in a report by the Epidemiology Network (Epi-Net) working group [25]. Third-generation cephalosporins resistant *E. coli*, and especially ESBL-producing *E. coli*, were monitored by the majority of ABR surveillance programmes in human, animal and food sectors, and appeared as a good candidate for integrated data analysis, hence complementing sectoral monitoring, as already suggested by ongoing One Health initiatives on antibiotic resistance, such as the WHO Tricycle protocol [26]. However, our study pointed out some major methodological differences between programmes, which make joint analysis challenging. ABU indicators used were also quite diverse within and between sectors, which could hinder data integration efforts and interoperability. ABR indicators in contrast, were more harmonised, but the interpretation criteria and antibiotic susceptibility testing standards differed. Those key-points should be addressed to ensure comparability of data across and within sectors in the future.

A few overlaps were identified in the French surveillance system, mainly in the human sector, where five programmes targeted ABR in healthcare facilities. These overlaps are due to older programmes at local or national levels that persisted when the programmes of the RéPias were initiated in 2018. There is a need to clarify the objectives of these overlapping programmes and their role within the overall surveillance system to improve its efficiency.

Conversely, we pointed out several gaps in the French surveillance system. First, the environmental sector was largely uncovered: we identified only one programme that complied with our definition of a surveillance programme. Other initiatives existed but were not sustainable at this stage. Structured national surveillance of antibiotic residues was limited to surface water and animal-derived food. No residues surveillance programme was identified in other important areas such as farm environments or wastewater treatment plants, although various research studies explored this issue [27,28]. This was not surprising as worldwide efforts towards environmental surveillance of ABR and antibiotic residues have recently started [29]. Still, the inclusion of the environmental sector in One Health approaches has been growing lately, as shown by the recent integration of the United Nations Environment Programme (UNEP) into the One Health antimicrobial resistance activities of the Quadripartite Alliance [30]. Of note, the European watch list for water surveillance targeted only a limited number of antibiotic classes [18]. There is a need to enlarge and strengthen the structuration of ABR and antibiotic residues surveillance in the environment, and to harmonise surveillance indicators being used in this sector, as recommended in the French national action plan for the environment and health [11].

Second, the coverage of the surveillance system could be further improved both in the human and animal sectors. Overseas territories were poorly represented in programmes with national geographic coverage. In the human sector, surveillance covered the three main populations of interest (healthcare facilities, long-term care facilities and the community). However, most human programmes focused on clinical samples reflecting suspicions of infections, with a lack of data on colonisation by antibiotic-resistant bacteria. This may underestimate spread of emerging resistance, e.g. carbapenemase-producing Enterobacterales, for which infection rates remain low in France, but dissemination is increasing [31].

In the animal sector, both healthy and diseased animals were covered by national ABR surveillance programmes, a situation still uncommon in Europe [32]. Additionally, several farm-level ABU surveillance programmes dedicated to selected livestock species complemented the overall surveillance of sales data. Yet, an important gap was a dedicated ABU-surveillance programme in companion animals. However, the upcoming implementation of the European Regulation 2019/6 on veterinary medicinal products, that covers both livestock and companion animals, should address this gap within a few years (by 2027 for horses and 2030 for dogs/cats [33]). To meet demands from this regulation, a new data collection system (called Calypso) will be implemented in France from 2023 onwards and collect antibiotic deliveries data per animal species; this may challenge the relevance and sustainability of the existing farm-level ABU surveillance programmes. Another gap in the animal sector is the lack of ABR surveillance in non-captive wild animals and aquaculture, although the RESAPATH programme already collects some antibiotic susceptibility testing data from fish production [34]. Additionally, antibiotic susceptibility testing in diseased animals was restricted to antibiotics authorised in veterinary medicine, which limits the assessment of the zoonotic exposure to ABR of human health relevance (e.g. resistance to carbapenems). The EARS-Vet initiative, launched during the EU-JAMRAI (eu-jamrai.eu), which intends to develop a European programme for surveillance of ABR in clinical pathogens of animals, recently proposed a panel of antibiotics of primary interest to both animal and human health [35].

A major strength of this study was the comprehensive approach we used, addressing ABR from a broad perspective including ABR, ABU and antibiotic residues in humans, animals and the environment, since these are closely connected. To our knowledge, no such overview is available elsewhere in the literature. By direct exchange with the coordinators of each programme, we are confident that our data are accurate and validated. We believe this mapping will be of interest to policymakers, as well as surveillance stakeholders, not only in France but also elsewhere since our methodology can easily be transferred to other countries and surveillance contexts.

Nonetheless, this work also had some limitations. While we collected detailed data on ABR/ABU indicators together with other information generated by each programme, getting access to the actual programme databases, e.g. to look at data formats, or thesaurus, was beyond the study scope. Hence, we were unable to evaluate the inter-operability of existing data. Additionally, our mapping only provided a snapshot of the surveillance system in 2021 and did not capture changes over time. Yet, the French ABR-related surveillance system appeared as an ever-evolving system, with several programmes and sub-systems that were launched, and others discontinued in recent years. For example, two large national meta-networks [36] funded through the French Priority Research Programme on ABR were started in November 2021: (i) the meta-network PROMISE aims to build a One-Health community of actors on ABR, to develop a joint data warehouse for ABR surveillance and to set up a national network for environmental surveillance of ABR, and (ii) the meta-network ABRomics-PF aims to build a platform for ABR multi-omics One Health data sharing. Those two meta-networks appear as excellent opportunities to further facilitate integration of surveillance programmes, and address some of the gaps identified in this study.

Our study was the first step to assess how the French surveillance system can operate in a One Health approach perspective. An in-depth investigation of existing collaborations between surveillance programmes as well as the main drivers for these is still under progress. Its results will help refine our practical recommendations to improve One Health surveillance of ABR in France. We believe that the approach including (i) identification and characterisation surveillance programmes, (ii) mapping the surveillance system to identify gaps and overlaps, and (iii) ultimately the evaluation of collaborations between programmes, presents an added value for ABR surveillance and will inspire other countries considering a gradual transition toward One Health surveillance of ABR.

## Conclusion

This first mapping and characterisation of the French surveillance system for ABR, ABU and antibiotic residues showed a resourceful and varied yet complex and fragmented system, involving multiple programmes. Overall, these programmes provide good coverage of key target populations in the human and animal sectors; however, some gaps were identified, notably in the environmental sector, which is largely uncovered. This study is an important step for future evaluation of the possibilities of One health ABR surveillance in France.

## Supplementary Data

44000 Supplementary material S1

## Supplementary Data

122000 Supplementary material S2
