# Erratum for Euro Surveill. 2023;28(22)

**DOI:** 10.2807/1560-7917.ES.2023.28.23.230608c

**Published:** 2023-06-08

**Authors:** Eurosurveillance editorial team

**Affiliations:** 1 European Centre for Disease Prevention and Control (ECDC), Stockholm, Sweden

In the article entitled ‘Towards One Health surveillance of antibiotic resistance: characterisation and mapping of existing programmes in humans, animals, food and the environment in France, 2021’ by Collineau et al., which was published on 1 June 2023, part of the text in the Figure footnote had been erroneously left out. The missing text was added on 5 June 2023. We apologise for any inconvenience this may have caused.

