# Supplementary material for: Towards One Health surveillance of antibiotic resistance: characterisation and mapping of existing programmes in humans, animals, food and the environment in France, 2021
Source: Euro Surveill. 2023 Jun 1;28(22):2200804. doi: 10.2807/1560-7917.ES.2023.28.22.2200804 (PMC10236929; doi:10.2807/1560-7917.ES.2023.28.22.2200804)
Supplement: Supplementary material S2 [file 2200804_SupplementaryMaterial_S2.pdf]

Supplementary material S2

This supplementary material is hosted by *Eurosurveillance* as supporting information alongside the article ” Towards One Health surveillance of antibiotic resistance: characterisation and mapping of existing programmes in humans, animals, food and the environment in France, 2021”, on behalf of the authors, who remain responsible for the accuracy and appropriateness of the content. The same standards for ethics, copyright, attributions and permissions as for the article apply. Supplements are not edited by *Eurosurveillance* and the journal is not responsible for the maintenance of any links or email addresses provided therein.

Guide used for interviews of the programmes’ coordinators for surveillance of antibiotic resistance, antibiotics use, and antibiotics residues, France, 2021 – the Surv1Health project

Role and activities of the interviewee in the surveillance programme

- What is your background/discipline?
- What is your current position?
- At which level are you working (national, regional, local)?
- What is your role in the surveillance programme?
- What activities do you achieve in the surveillance programme?

Context of the creation of the surveillance programme

- When was the programme created? Is it an evolution of a pre-existing programme?
- What was(were) the reason(s) for creating this programme? Please describe the political, economic and epidemiological context when the programme was created.

Objectives of the programme

- What is(are) the objective(s) of the programme: early warning, trends monitoring, expertise, etc.?

Laboratories

- What is the enrolment process for laboratories willing to join the programme?
- Is there a quality control requirement for joining the programme?
- What step(s) of data curation/harmonization is(are) operated by the laboratories?
- Are you working in collaboration with a national reference laboratory? If yes, which one and for which activities?

Data production

- What kind of data are being collected (e.g. bacterial strains, epidemiological data, metadata) by the programme?
- Who is responsible for data management and data storage?
- How are raw data curated and validated?
- How is antimicrobial susceptibility testing being performed? Please describe shortly the techniques and standards being used.
- What volumes of data are you collecting?

Indicators

- What are the main indicators generated by the programme?

- How often and via which media channels are they published? Who are the main recipients / target audiences?
